# Trimetazidine mitigates high glucose-induced retinal endothelial dysfunction by inhibiting PI3K/Akt/mTOR pathway-mediated autophagy

**DOI:** 10.1080/21655979.2022.2048993

**Published:** 2022-03-08

**Authors:** Qingsong Yang, Sizhen Li, Zixiu Zhou, Xiaodong Yang, Yating Liu, Kuanxiao Hao, Min Fu

**Affiliations:** Nanjing Tongren Eye Center, Nanjing Tongren Hospital, School of Medicine Southeast University, Nanjing, P.R. China

**Keywords:** Trimetazidine, autophagy, retinal endothelial dysfunction, PI3K/Akt/mTOR pathway

## Abstract

Trimetazidine (TMZ), as a metabolic regulator, has been widely testified to exhibit positive therapeutic effects on various disease models, but its role in diabetic retinopathy has not been reported. Therefore, this study was designed with the purpose of exploring the effects of TMZ on high-glucose (HG)-induced retinal endothelial dysfunction and its underlying mechanism. To establish DR model in vitro, 30 mM glucose was applied to induce human retinal endothelial cells (HRECs). Cell proliferation, invasion, and migration were examined by means of Cell Counting Kit-8, transwell, and wound healing assays, respectively. The tubule formation experiment was used to test the tubulogenesis ability and fluorescein isothiocyanate (FITC)-albumin was utilized to measure the permeability of monolayer HRECs. In addition, immunofluorescence and Western blot were employed to detect protein expression. Compared with the HG-induced group, TMZ concentration dependently inhibited the proliferation, migration, and angiogenesis of HG-induced HRECs, decreased the permeability of monolayer HRECs, and increased the protein expression levels of Claudin-5 and VE-cadherin. In addition, TMZ intervention increased the expression of p-PI3K, p-AKT, and p-mTOR but decreased the expression of LC3I, LC3II, and Beclin 1, which were then partially reversed by P13 K inhibitor (LY294002). Moreover, the autophagy agonist rapamycin (RAPA) was also testified to reverse the inhibitory effects of TMZ on the proliferation, migration, and angiogenesis of HG-induced HRECs. In summary, TMZ inhibited excessive autophagy by activating PI3K/Akt/mTOR pathway, thereby improving retinal endothelial dysfunction induced by HG.

## GRAPHICAL ABSTRACT

**Figure:**
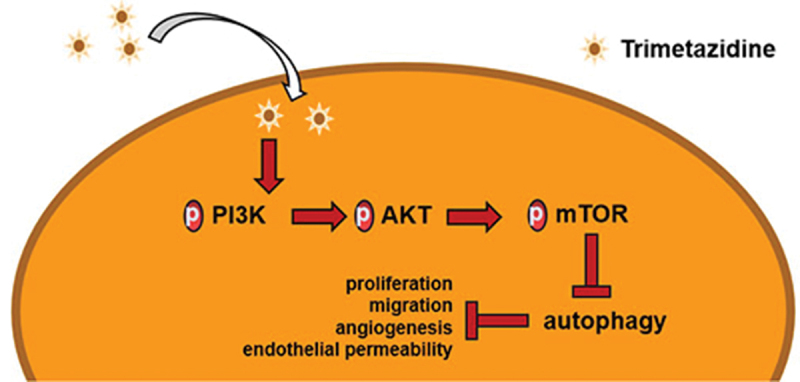

## Introduction

Diabetic retinopathy is a diabetic microvascular complication that gradually emerges with the development of chronic progressive diabetes [1], and is characterized by the loss of peripheral cells, the increase of new capillaries and the enhancement of blood-retinal barrier permeability [2]. Angiogenesis is an important factor in the development of diabetic complications [3]. During the pathogenesis, the increase of vascular endothelial growth factor (VEGF) can induce retinal endothelial permeability and proliferation, thereby promoting retinal angiogenesis and ultimately developing proliferative diabetic retinopathy [4]. In diabetes or hyperglycemia, the increased permeability of retinal endothelial cells disrupts the blood-retinal barrier, resulting in retinal hemorrhage, exudation, and detachment [5]. Retinal endothelial cell dysfunction is the main pathological process of diabetic retinopathy [6]. Therefore, the aim of this study was to explore the effects of TMZ on HG-induced retinal endothelial dysfunction and its underlying mechanisms.

TMZ is an anti-angina pectoris drug, which could improve the energy metabolism of cells under the condition of hypoxia or ischemia and prevent the decrease of intracellular ATP level, thus ensuring the normal function of ion pump and maintaining the stability of intracellular environment [7,8]. In recent years, the role of TMZ has been widely reported. For example, a previous study demonstrated that TMZ may protect cardiomyocytes from hypoxia/reoxygenation-induced injury by regulating autophagy through upregulating HMGB1 expression [9]. Wu et al. found that TMZ inhibited autophagy by activating AKT/mTOR pathway, thus protecting myocardial ischemia/reperfusion injury [10]. The combination of ischemic post-treatment and TMZ could effectively reduce endoplasmic reticulum stress and mitochondrial damage in kidney [11]. Furthermore, a recent study has demonstrated that TMZ could improve retinal lipid peroxidation and histopathological changes caused by ischemia-reperfusion injury [12]. TMZ also exhibits anti-fibrosis effects by reducing reactive oxygen species and collagen production in myocardial fibroblasts [13]. However, to the best of our knowledge, its role in diabetic retinopathy has not been reported.

A large number of studies have shown that autophagy is closely related to endothelial cell dysfunction. For example, aspirin inhibited autophagy by regulating beclin-1 phosphorylation through Vps15 scaffold, and reversed endothelial dysfunction induced by Estrogen [14]. Physcion 8-O-bglucopyranoside regulated autophagy by activating AMPK/SIRT1 signaling pathway, thereby protecting human umbilical vein endothelial cells (HUVECs) from oxidized low-density lipoprotein-induced injury [15]. Endothelial dysfunction caused by palmitic acid-induced autophagy was ameliorated by downregulating ROS production with NOX4 inhibitors [16]. Notably, a previous study showed that Naringin inhibited autophagy by activating the PI3K-Akt-mTOR pathway and improved HUVECs dysfunction under HG/fat stress [17]. It is well known that PI3K-Akt-mTOR pathway, as a key regulator of autophagy, is involved in the initiation and promotion of various pathological diseases [18,19]. In general, the activation of PI3K-Akt-mTOR pathway promotes cell proliferation, migration, and invasion, while the inhibition of this pathway leads to autophagy [20,21].

Therefore, in this study we hypothesized that TMZ could ameliorate HG-induced retinal endothelial dysfunction by inhibiting autophagy mediated by PI3K/Akt/mTOR pathway, so as to find new therapeutic drugs for diabetic retinopathy.

## Materials and methods

### Cell culture and treatment

Human retinal endothelial cells (HRECs) were purchased from Ningbo Mingzhou Biotechnology Co., LTD (cat. No. MZ-1174). Cells were cultured in Endothelial Cell Medium (Hyclone, USA, cat. No 1001) supplemented with 10% Fetal Bovine Serum (Gibco; USA, cat. No 10,091,141) at 37°C with 5% CO_2_. The cells in the logarithmic growth phase were incubated with normal glucose (5.5 mM, Sigma-Aldrich, cas. No 50–99-7), high glucose (HG, 30 mM, Sigma-Aldrich, cas. No 50–99-7) [22], LY294002 (10 μM, Sigma-Aldrich, cas. No 934,389–88-5) [17] and Rapamycin (50 μM, Sigma-Aldrich, cas. No 53,123–88-9) at 37°C for 24 h, respectively.

### Cell counting kit-8 (CCK-8) assay

HRECs with a density of 5 × 10^3^ cells per well were inoculated in a 96-well cell culture plate and incubated overnight at 37°C with 5% CO_2_. Following the treatment with different concentrations of drugs for 24 h, 10 µl CCK-8 solution (Vazyme, cat. No A311-01/02) was added to each well and the cells were incubated at 37°C with 5% CO_2_ for 4 h. Absorbance was measured at 450 nm using a Varioskan™ LUX Multi-function microplate reader (Thermo Fisher Scientific, Inc.).

### Detection kit

Click-it™ EdU (5-Ethynyl-2’-deoxyuridine) Cell Proliferation Imaging Kit (Thermo Fisher Scientific, Inc. C10337) was used to detect cell proliferation activity [23]. VEGF expression levels were determined using the VEGF Human ELISA Kit (Solarbio, SEKH-0052-96) according to the manufacturer’s agreement [24].

### Cell permeability detection

HRECs (1x10^5^ cells/well) were inoculated in the upper chamber of 24-well transwell plates (Corning, Inc.) with 8 μm pore inserts for 12 h, and a complete media (1.5 ml) was added to the lower chamber. Non-adherent cells were washed with PBS and fresh ECM medium was added. Subsequently, FITC-albumin (Sigmal-Aldrich, 1 mg/ml) was added to the apical compartment and then incubated for 1 h at 37°C with 5% CO_2_. Media was removed from the lower chamber and the fluorescence intensity of each sample was measured with excitation wavelength of 490 nm and emission wavelength of 525 nm by a multifunctional plate reader (Molecular Devices) [25].

### Immunofluorescence staining

Cell slides were placed at the bottom of the 24-well plate. Cells (1x10^5^ cells/well) were inoculated on the slides and cultured at 37°C with 5% CO_2_ until cell fusion reached 80%. After being gently washed with PBS, the fixation of cells with 4% paraformaldehyde (Sigma-Aldrich, USA) was performed for 15 min, followed by infiltration with 0.1% Triton X-100 (Sigma-Aldrich, USA) for another 15 min. Samples were blocked with 5% appropriate serum/1 × PBS at 37°C for 30 min followed by incubation with following primary antibodies for 2 h at 4°C: claudin-5 (Abcam; 1:5,000; ab131259), VE-cadherin (Abcam; 1:1,000; ab33168) and Beclin 1 (Abcam; 1:1,000; ab207612). After washing with 1x tris buffered saline tween (TBST), the samples were incubated with suitable Alexa Fluor-conjugated secondary antibody (Abcam, 1:1,000; ab150077) at 37°C for 1 h. The samples were washed with PBS and then DAPI (4′,6-diamidino-2-phenylindole) staining solution (Beyotime, cat. No. C1005) was applied for staining for 5 min. After staining, the cells were washed with PBS for three times and images were acquired under fluorescence microscope (Thermo Fisher Scientific, Inc; magnification, x200) [26].

### Wound healing assay

HRECs (1x10^6^ cells/well) were seeded in a 12-well plate and incubated in a cell incubator at 37°C with 5% CO_2_ until the cells adhered. When the cell confluence reached 70%–80%, the cell culture medium was replaced with serum-free ECM and the cells were cultured overnight, Then, a 200 µl pipette tip was applied to scratch the cell monolayer. After the wash with PBS, the cells were incubated in a cell incubator for 48 h. Finally, the wounds were observed using a EVOS™ M7000 imaging system (Thermo Fisher Scientific, Inc; magnification, x100). Cell migration was quantified as follows: (0 h scratch width – scratch width following culturing)/0 h scratch width [27].

### Transwell assay

To verify HREC invasion, 24-well Transwell plates (Corning, Inc.) with 8 μm pore inserts were coated with Matrigel (Solarbio Inc.) at 37°C for 30 min. Cells (3x10^4^ cells/ml) in serum-free ECM medium were plated into the upper chamber and ECM medium supplemented with 10% FBS was added to the lower chamber, following which was the incubation for 24 h at 37°C with 5% CO_2_. After the removal of noninvasive cells, the cells were fixed with 4% formaldehyde at room temperature for 20 min, and stained with 0.1% crystal violet solution at room temperature for 30 min. Five fields were randomly selected to observe the stained cells using a EVOS™ M7000 imaging system (Thermo Fisher Scientific, Inc; magnification, x100) [28].

### Tube formation assay

24-well culture plate, 200 µL pipette tips, and matrigel matrix glue were placed at 4°C overnight. Subsequently, about 200 µL of matrix gel was sucked into the 24-well plate, and placed in a cell incubator to gel for 30 min. Before that, the culture medium of HRECs in each group was changed to serum-free culture medium for 24 h. Then the cells were digested with trypsin-EDTA solution (Beyotime, cat. no. C0201) to prepare a single-cell solution. The culture medium containing 300 µL serum and 10 µL single-cell suspension was added to each well, and the culture was continued for 10 h. Finally, photographs were taken with an inverted microscope (ZEISS, Axio Vert.A1, magnification, x40).

### Western blotting

The cells were washed with pre-cooled PBS for three times and then cleaved with RIPA lysis buffer (Beyotime, cat. no. P0013C) for 30 min on ice. Then cell lysates were collected and centrifuged (300 x g) at 4°C for 15–20 min. Protein supernatant in different groups were transferred to Eppendorf tubes. Total proteins were quantified using the compat-Able™ BCA protein assay kit (Thermo Fisher Scientific, Inc; cat. No. 23,229). The proteins that added into each group was 40 µg and separated by 10% SDS-PAGE. Then, the proteins were transferred to PVDF membrane (Beyotime, cat. no. FFP24) and sealed with 5% defatted milk powder at room temperature for 4 h. After washing with 1x TBST for 3 times, the membranes were cultured overnight with the following primary antibodies (all purchased from Abcam) at 4°C: Anti-p-PI13 K (1:1,000; cat. no. ab278545), anti-p-AKT (1:1,000; cat. no. ab38449), anti-p-mTOR (1:1,000; cat. no. ab109268), anti-PI13 K (1:1,000; cat. no. ab32089), anti-AKT (1:1,000; cat. no. ab8805), anti-mTOR (1:1,000; cat. no. ab134903), anti-LC3I/II (1:1,000; cat. no. ab63817), anti-Beclin 1 (1:1,000; cat. no. ab207612), anti-p62 (1:1,000; cat. no. ab240635) anti-VEGF (1:1,000; cat. no. ab32152) and anti-GAPDH (1:1,000; cat. no. ab8245). After primary incubation, the membranes were incubated with goat anti-rabbit horseradish peroxidase conjugated IgG secondary antibody for 4 h at room temperature, and the protein bands were observed using enhanced chemiluminescence reagent (Thermo Fisher Scientific, Inc). Protein expression levels were semi-quantified using ImageJ software (version 1.8.0; National Institutes of Health) with GAPDH serving as the loading control [29].

### Statistical analysis

The measured data were expressed by mean ± standard deviation from ≥3 independent experiments and GraphPad Prism 8.0 software (GraphPad Software, Inc.) was used to plot the figures. Student’s t-test was performed for comparisons between two groups, and one-way ANOVA followed by Tukey’s post hoc test was used for comparisons among multiple groups. P < 0.05 was considered to indicate a statistically significant difference.

## Results

This study investigated the role of TMZ on the dysfunction of retinal endothelial cells induced by HG, and its potential molecular mechanism. First, we evaluated the effects of TMZ on proliferation, invasion, migration, and angiogenesis of human retinal endothelial cells. We found that TMZ concentration dependently inhibited the abnormal proliferation, invasion, migration, and angiogenesis of HRECs induced by HG. Subsequently, we further evaluated the effects of TMZ on cell membrane permeability and autophagy of HRECs. The results showed that TMZ inhibited excessive autophagy by activating PI3K/Akt/mTOR pathway, and reduced the permeability of cell membrane, thus improving the retinal endothelial dysfunction induced by HG, suggesting that TMZ might be a potential drug candidate for treating diabetic retinopathy.

### TMZ inhibited the proliferation and migration of HRECs induced by HG

**Figure 1.**
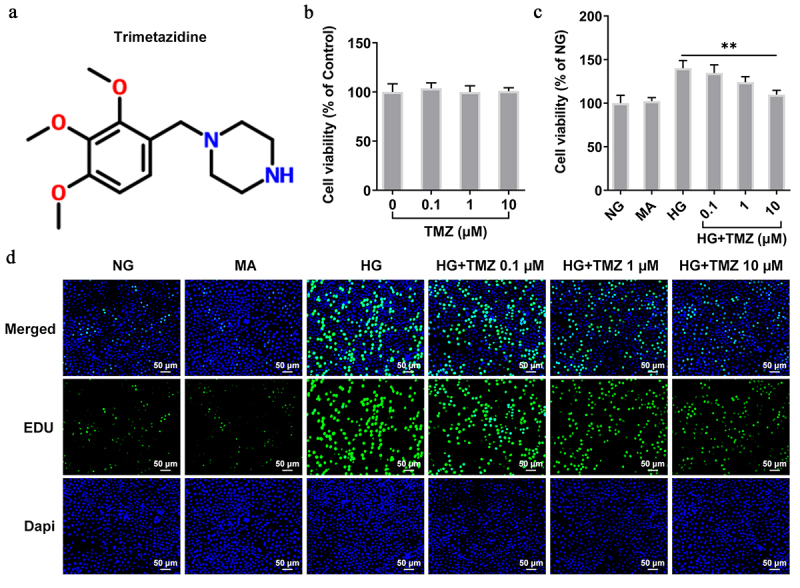
TMZ inhibited the proliferation of HRECs induced by HG.

CCK-8 experiment detected the effects of TMZ (Figure 1(a)) with different concentrations (0.1 µM, 1 µM, and 10 µM) on HRECs cell viability. The results showed that TMZ at these three concentrations had no obvious effects on HRECs cell viability, which indicated the good biocompatibility of TMZ (Figure 1(b)). Subsequently, 5.5 mM (normal glucose, NG) and 30 mM glucose (high glucose, HG) were incubated with HRECs for 24 h at 37°C to simulate normal blood glucose and HG environment, respectively. Interventions were performed using different concentrations of TMZ (HG + TMZ 0.1 µM, HG + TMZ 1 µM, and HG + TMZ 10 µM), in which 10% mannitol (MA) was used as negative control. Compared with the control group, the HG induction significantly promoted the cell proliferation. Nevertheless, TMZ intervention inhibited the proliferation of HG-induced HRECs in a concentration-dependent manner in contrast with that in HG group (Figure 1(c)), which was consistent with the detection by EDU (Figure 1(d)).

(a) Chemical formula for TMZ. (b) Cell Counting Kit-8 assay detected the effects of TMZ with different concentrations (0.1 µM, 1 µM and 10 µM) on HRECs viability. (c) Effects of TMZ on HRECs viability induced by HG. (d) EDU examined the effects of TMZ on HRECs proliferation induced by HG. **P < 0.01 vs. HG. TMZ, Trimetazidine; HRECs, Human retinal endothelial cells; EDU, 5-Ethynyl-2’-deoxyuridine; HG, high glucose.

### TMZ inhibited HRECs migration and angiogenesis induced by HG

**Figure 2.**
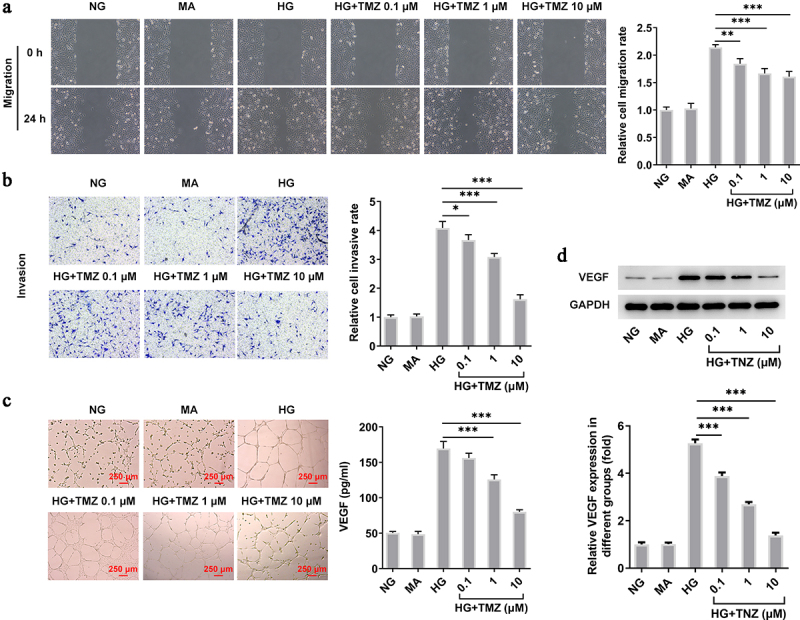
TMZ inhibited migration, invasion, and angiogenesis of HG-induced HRECs.

Consistent with the above groupings and conditions, we continued to detect the influence of TMZ on HRECs migration and invasion in HG condition. As shown in Figure 2(a,b), HG induction significantly enhanced the cell migration and invasion while TMZ effectively prevent the abnormal reaction of HRECs in such HG environment. Subsequently, we tested the tube forming ability of HRECs under the same treatment conditions. Also, TMZ alleviated the enhanced tubulogenesis ability of HG-induced HRECs in a concentration-dependent manner, the expression of VEGF protein in HRECs showed the same result (Figure 2(c,d)). Therefore, the above results suggested that TMZ may have the potential to slow down the progression of diabetic retinopathy.

(a)Wound healing and (b) Transwell assay were performed to test the effects of TMZ on the migration and invasion of HERCs induced by HG, respectively. (c) Tubule formation assay and VEGF content were employed to detect the tube-forming ability of HERCs. *P < 0.05, **P < 0.01 and ***P < 0.001 vs. HG. HRECs, Human retinal endothelial cells; VEGF, vascular endothelial growth factor; HG, high glucose.

### TMZ decreased the permeability of HG-induced HRECs, and increased the expression of Claudin-5 and VE-cadherin proteins

**Figure 3.**
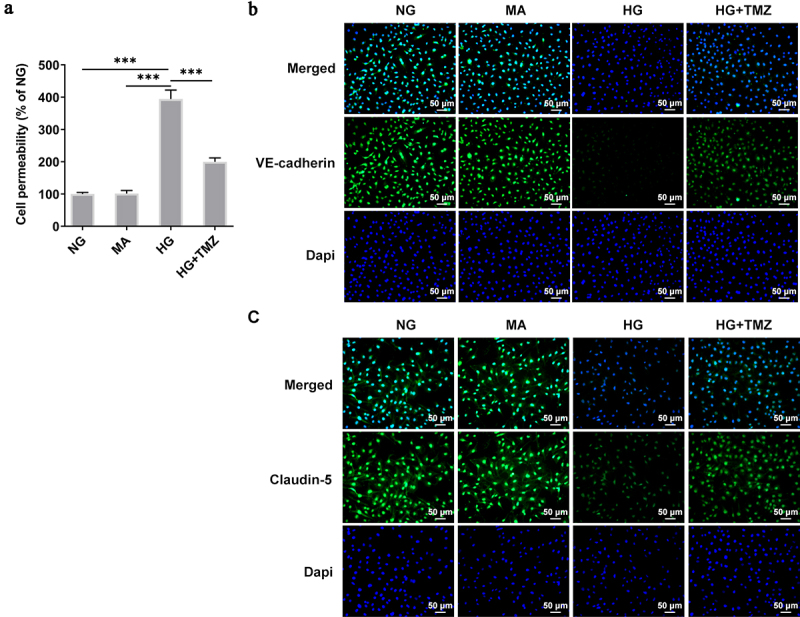
TMZ decreased the permeability of HRECs cells induced by HG, and increased the expression of Claudin-5 and VE-cadherin proteins.

Fitc-albumin was used to detect the permeability of monolayer HRECs. Compared with cells under normal blood glucose, the permeability of monolayer cells induced by HG significantly increased. However, under the same conditions, TMZ 10 µM reduced the permeability of HG-induced of HRECs (Figure 3(a)). In addition, Ve-cadherin and Claudin-5 are key components of adhesion and tight endothelial junctions, respectively, and play important roles in the barrier function of various vascular endothelial cells [30]. It was shown that VE-cadherin can control the expression of Claudin-5 by blocking nuclear accumulation of FoxO1 and β-catenin [31]. Therefore, we observed the expression of Claudin-5 and VE-cadherin by immunofluorescence. Figure 3(b,c) show that HG environment inhibited the expression of claudin-5 and VE-Cadherin proteins in HREC. However, TMZ greatly enhanced the expression of Claudin-5 and VE-Cadherin proteins.

(a) FITC-albumin was used to detect the permeability of monolayer HRECs. The expression levels of VE-Cadherin (b) and Claudin-5 (c) proteins in HRECs were detected by immunofluorescence. ***P < 0.001 vs. NG, MA, or HG. HRECs, Human retinal endothelial cells; NG, normal glucose; MA, mannitol; HG, high glucose.

### TMZ inhibited autophagy mediated by PI3K/Akt/mTOR pathway

**Figure 4.**
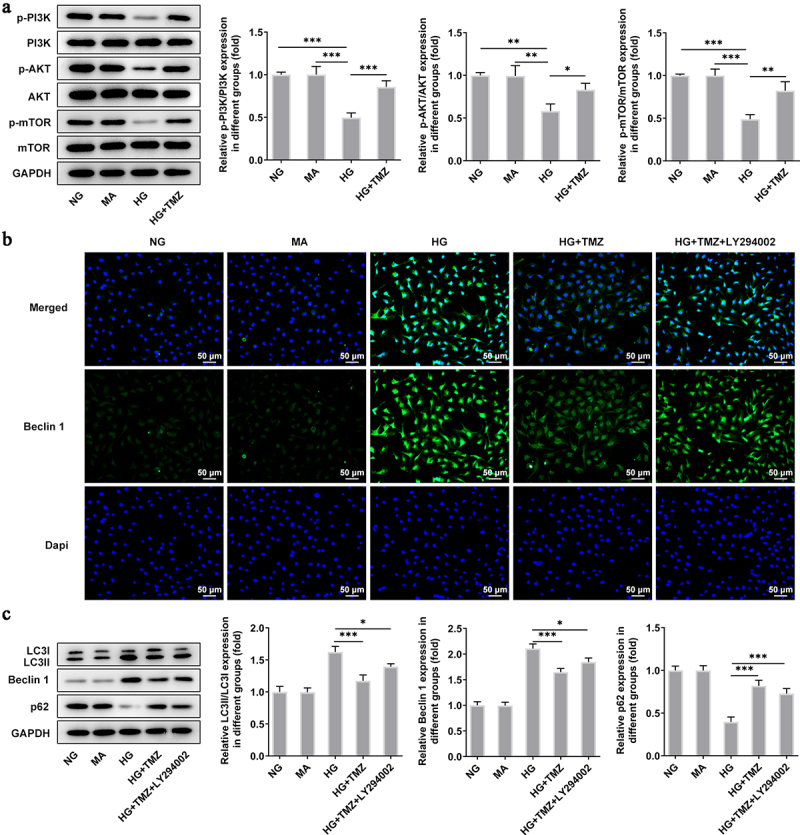
TMZ inhibited autophagy mediated by PI3K/Akt/mTOR pathway.

We analyzed the effect of TMZ on the autophagy-mediated by PI3K/Akt/mTOR pathway in retinal endothelial cell dysfunction induced by HG [32,33]. The results of western blot showed that PI3K/Akt/mTOR pathway was inhibited in the high-glucose environment, which was manifested by the decreased levels of p-PI3K, p-Akt and p-mTOR proteins. Notably, the inhibition of PI3K/Akt/mTOR pathway caused by HG was obviously reversed with 10 μM TMZ intervention (Figure 4(a)). Subsequently, the expression of autophagy-related proteins (LC3I, LC3II, Beclin-1 ,and P62) were also detected by Western blot, and the results indicated that the increased expression of LC3II, LC3I, and Beclin-1 proteins as well as decreased level of P62 protein in HG-induced HRECs were significantly reversed by TMZ pretreatment. After the administration of LY294002, an inhibitor of P13 K, the effects of TMZ treatment on autophagy-related proteins were partially abolished. (Figure 4(c)). In order to observe the protein levels more intuitively, we adopted immunofluorescence to detect Beclin-1, and obtained consistent results (Figure 4(b)).

(a) Expression levels of PI3K/Akt/mTOR pathway-related proteins were measured via western blotting. (b) The expression level of Beclin 1 was detected by immunofluorescence. (c) Western blot was conducted to assay the expression of autophagy-related proteins. *P < 0.05, **P < 0.01 and ***P < 0.001 vs. NG, MA, or HG. NG, normal glucose; MA, mannitol; HG, high glucose.

### Rapamycin (RAPA) reversed the ameliorative effects of TMZ on retinal endothelial cell dysfunction induced by HG

**Figure 5.**
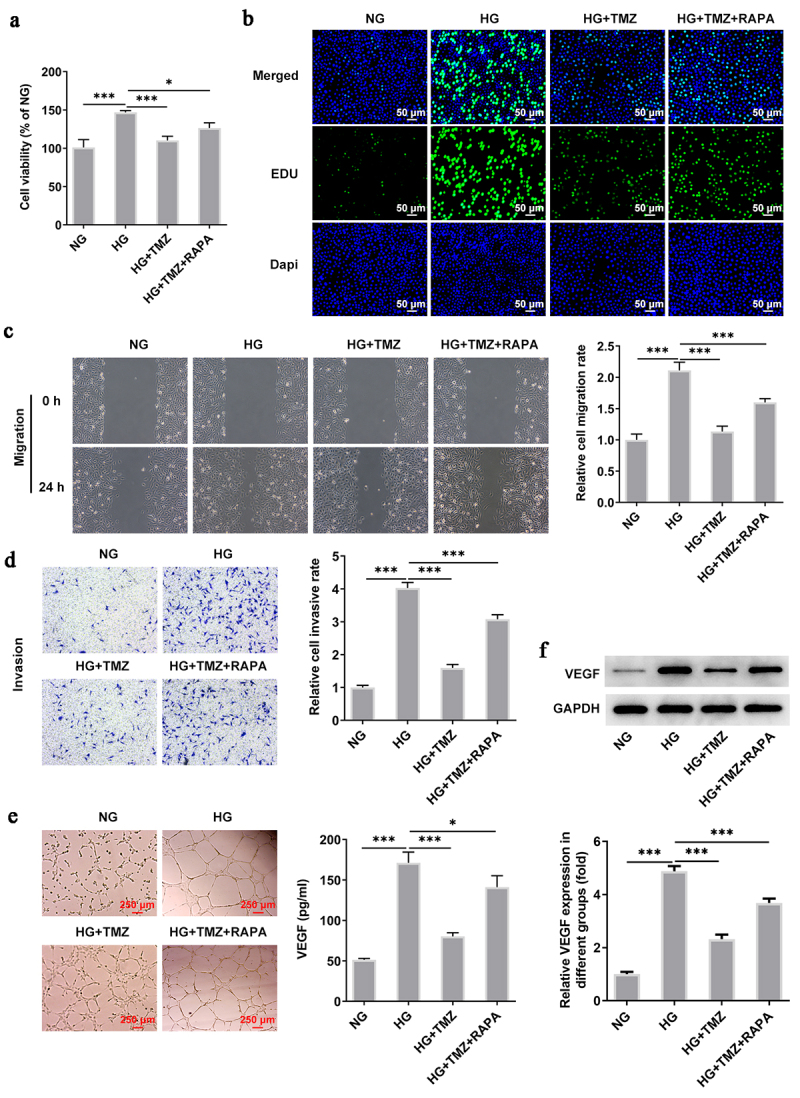
RAPA reversed the inhibitory effect of TMZ on HRECs proliferation, migration, invasion and angiogenesis induced by HG.

**Figure 6.**
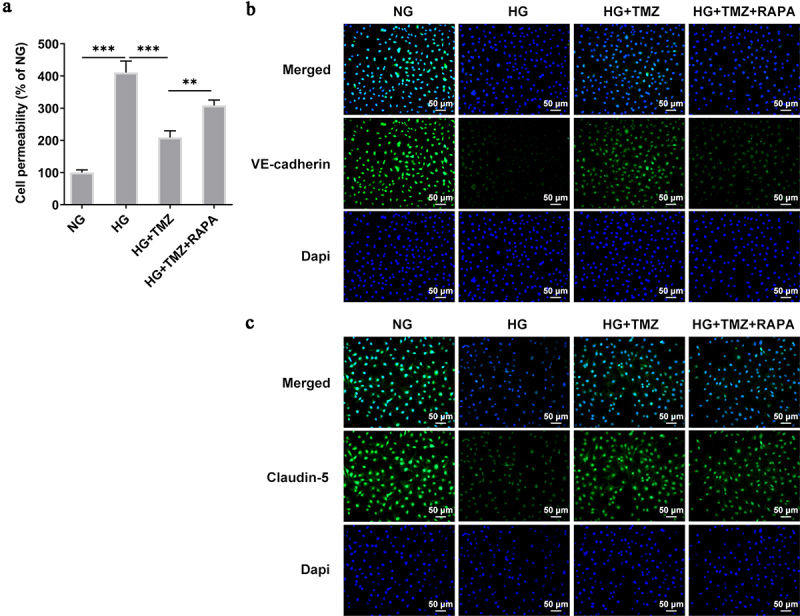
RAPA reversed the inhibitory effect of TMZ on HRECs proliferation, migration, invasion and angiogenesis induced by HG.

Following experiments were conducted with the aim of further testifying that TMZ alleviates HG-induced retinal endothelial cell dysfunction by inhibiting autophagy mediated by PI3K/Akt/mTOR pathway. Rapamycin (RAPA), an autophagy agonist, was introduced to reverse the ameliorative effects of TMZ on retinal endothelial cell dysfunction induced by HG. Compared with HG + TMZ group, the addition of RAPA partially abolished the inhibitory effects of TMZ on the proliferation (Figure 5(a,b)), migration (Figure 5(c)) and invasion (Figure 5(d)) of HG-induced, as well as the increase of tubulogenesis ability (Figure 5(e,f)) and cell permeability (Figure 6(a)). At the same time, the promoted expression of intracellular claudin-5 and VE-cadherin proteins in HG-induced HRECs caused by TMZ treatment was diminished after RAPA administration (Figure 6(b,c)). These results suggested that TMZ alleviated HG-induced retinal endothelial dysfunction may be through the inhibition of autophagy which was mediated by PI3K/Akt/mTOR pathway.

Representative images of (a) wound healing analysis, (b) Transwell analysis and (c) tubule formation experiments, and their corresponding quantization levels. *P < 0.05 and ***P < 0.001 vs. NG, MA, or HG. NG, normal glucose; MA, mannitol; HG, high glucose. RAPA, rapamycin; HRECs, Human retinal endothelial cells.

FITC-albumin was used to detect the permeability of monolayer HRECs. The expression levels of VE-Cadherin (b) and Claudin-5 (c) proteins in HRECs were detected by immunofluorescence. **P < 0.01 and ***P < 0.001 vs. NG, or HG + TMZ. TMZ, Trimetazidine; HRECs, Human retinal endothelial cells; RAPA, rapamycin; NG, normal glucose; HG, high glucose.

## Discussion

In the present study, we investigated the effects of TMZ on HG-induced dysfunction of HRECs in an *in vitro*model. Through the establishment of HRECs pathological model with 30 mM glucose as inducer, we found that HG induction contributed to abnormal proliferation and migration of HRECs, and improved the angiogenesis ability of HRECs as well as the permeability of monolayer cells. The appearance of these early features of microvascular lesions suggested the successful establishment of the model [34–36]. Notably, TMZ intervention significantly improved HG-induced HRECs dysfunction in a concentration-dependent manner. In addition, our results showed that TMZ could activate PI3K/Akt/mTOR pathway and inhibit autophagy level under HG stimulation, while these effects were significantly eliminated by PI3K inhibitor (LY294002) which could specifically block PI3K/Akt/mTOR pathway [37]. RAPA, an autophagy agonist, reversed the ameliorative effects of TMZ on HG-induced retinal endothelial dysfunction. In summary, we revealed that TMZ could effectively inhibit HG-induced HRECs dysfunction and excessive autophagy. Furthermore, to our knowledge, this study was the first to demonstrate that TMZ inhibited excessive autophagy through the PI3K/Akt/mTOR pathway in an *in vitro*model of diabetic retinopathy, thereby improving retinal endothelial dysfunction.

It is commonly acknowledged that PI3K/AKT/mTOR pathway plays a crucial role in many cell biological processes, including the regulation of cell proliferation, differentiation, apoptosis, and migration [37,38]. As a pivotal molecule in regulating growth and metabolism, mTOR has been shown to negatively regulate autophagy activity [39,40]. A previous study has shown that TMZ regulated autophagy by up-regulating the expression of HMGB1, thereby protecting against hypoxia/reoxygenation-induced myocardial cell injury [9]. Wu et al. demonstrated that TMZ inhibited autophagy by activating AKT/mTOR pathway, thus protecting from myocardial ischemia/reperfusion injury [10]. In our study, compared with the HG group alone, TMZ treatment obviously decreased the protein expression of LC3-I, LC3-II, and Beclin1but increased p62 expression, which were then reversed by LY294002. Furthermore, TMZ up-regulated the expression of p-PI3K, p-Akt, and p-mTOR, leading to the down-regulation of autophagy level. Consequently, our results indicated that TMZ inhibited autophagy level by activating PI3K/Akt/mTOR pathway, which was similar with the results found by Wu et al.

In previous studies, inhibition of autophagy effectively improved endothelial dysfunction. For example, naringin inhibited autophagy by activating PI3K-Akt-mTOR pathway and improved HUVECs dysfunction under HG/fat stress [17]. Aspirin suppressed autophagy by modulating beclin-1 phosphorylation via Vps15 scaffold and reversed estrogen-induced endothelial dysfunction [14]. By down-regulating ROS production with NOX4 inhibitors, endothelial dysfunction caused by palmitic acid induced-autophagy can be improved [16]. The above results may be partially attributed to the important role of autophagy in angiogenesis. One study showed that TMEM16A reduced vascular remodeling by inhibiting the occurrence of autophagy, indicating that autophagy was positively correlated with angiogenesis [41]. Another study also found that autophagy played a key role in the neovascularization of retinal vascular endothelial cells, and targeting autophagy could significantly inhibit the formation of new blood vessels [42]. In this study, TMZ apparently inhibited the cell proliferation, migration, invasion, and angiogenesis induced by HG, decreased the permeability of monolayer cells, and increased the expression of Claudin-5 and VE-cadherin proteins, while autophagy agonist RAPA reversed these positive effects. In conclusion, the mechanism of TMZ in improving retinal endothelial dysfunction induced by HG is related to the inhibition of excessive autophagy, which is consistent with the above-cited reports.

Although we first proposed the positive role and mechanism of TMZ in diabetic retinopathy, the results we found were only supported in *in vitro* assays, and the verification in *in vivo* experiments will be the focus of our further research. Furthermore, it is indispensable to further expand the concentration range of TMZ to determine whether TMZ consistently improves retinal endothelial cell dysfunction in a concentration-dependent manner. In addition, it should be pointed out that the protective mechanism of TMZ might not only be related to the activation of PI3K/Akt/mTOR pathway, but other pathways remain to be further verified.

## Conclusion

In summary, we demonstrated that TMZ inhibited excessive autophagy by activating PI3K/Akt/mTOR pathway *in vitro*, thereby improving retinal endothelial dysfunction induced by HG. The interaction between TMZ and autophagy provides a new strategy for the prevention of diabetic retinopathy, suggesting that TMZ may be a potential drug candidate for the treatment of diabetic retinopathy.

## Data Availability

The datasets used and/or analyzed during the current study are available from the corresponding author on reasonable request.
